# Kali Carb,—Rheumatism

**Published:** 1881-03

**Authors:** E. W. Berridge

**Affiliations:** London


### KALI CARB.—RHEUMATISM.

E. W. BERRIDGE, M. D., LONDON.

1878, JULY 22d. Mr. ——, Æt forty-two. Seven years ago had the gout for nine weeks, since that attack has been told that his heart was "weak" but not "diseased." Three months ago had another attack in left foot; about sixteen days ago, after cold bath, this went to stomach, six days ago the heart was attacked. Has taken *Calomel*, *Pil. Rhei Co.*, *Sodæ Carb.*, *Vini Colch.*, *Aquæ Chlor.*, *Sodæ Salicyl.* (of course!) *Cinchona*, *Zingiber*, *Hydrarg. C. Creta*, *Hyos.*, and turpentine applications over heart: the curative effect of all this "scientific" and "rational" (!) treatment was *Nil.*

PRESENT STATE: Pain like a knife going into heart, *worse between* 3 *and* 4 *A.M.*, *when he generally wakes up with it*, *with fear of death*, attacks last an hour; last night some aching in stomach which is tender to touch; costive for three months; a systolic murmur loudest at apex; for two weeks has had pressure on heart, on leaning forward or carrying anything in left hand. The italicised condition pointed especially to *kali carb.* which also corresponded well with the other symptoms; I gave one dose of *kali c.* 3 C.M. (Fincke) in the morning. July 24th. Much better for last two nights, especially last night; did not on either occasion wake with the pain; yesterday had the pain in the heart about noon and in the afternoon, worse between 7 and 8 P. M., but less severe than before; the pain was shooting from below upward, he had not noticed the direction before; the last attack continued for forty-five minutes, with less fear of death; the stomach symptoms returned last evening; systolic murmur less; costiveness unchanged.

July 25th. Last night was not so good, awoke several times with a little pain in heart; had been pretty well during the day but became worse between 7 and 8 P. M.; to-day very little pain till about 1.45 P. M. when he had sharp pains in heart and again about 7 P. M; the pain in foot returned last night and has continued; it is in the ball of the great toe which is tender and burns; for the last two days much less costive than for the last three months; systolic murmur less; tenderness over apex of heart; the pressure over heart on leaning forward or carrying with left hand, continues.

July 26th. Had a better night; foot rather more painful; heart less painful and less tender. July 29th. Has slept very well; has had very little sharp pain at heart and not nearly so severe; has a dull, heavy feeling there, with tenderness; stomach pain not nearly so severe; the foot was very bad on 27th and 28th, but is much better to-day; systolic murmur nearly gone; rather more costive. Aug. 1st. No more shooting pain in heart, very little dull pain there; no return of stomach pain; sleep good; foot much better; no swelling, little redness or pain; no systolic murmur or tenderness at heart; still costive. Aug. 5th. Only a little dull pain at heart; foot a little more troublesome; stomach a little more tender to touch; sleep good; costive. Aug. 12th. No more pain at heart or stomach; foot still a little painful and red, but no swelling; much less costive.

Aug. 17th. Has had *slight* dull pain and tenderness at heart, and at times sharp pain; stomach well; pain still in arch of foot, toe nearly well; bowels natural; has had pain in the left hand (weather damp).

Aug. 22d. No pain at heart since 18th, no tenderness, foot better, hand well, bowels natural. Aug. 31st. Heart keeps well, only little pain in foot, bowels natural.

Sept, 7th. Of all the old symptoms there is only a slight pain in foot. About six times in the last three weeks, has had a feeling of pins and needles in both hands, usually on waking; last night woke with it in left hand, the fingers of which were drawn together over each other, but not bent; to-day this sensation extends up to shoulder, (effect of *kali c.*?). Sept. 11th. The above new symptoms have not returned since the 8th.

For two days has felt dull pain at heart and later on, shooting; these going downward toward left side, waking him before daybreak; shooting in heart when carrying hand-bag in left hand; to-day stomach is tender to touch; irritable for two or three days; worried by the children playing about, which was not the case in the former attack; heart sounds normal; sharp pain at heart on deep inspiration; the shooting pain is better when lying on the right side, worse lying on left side. On the 9th, the day before this relapse, he went out fishing and drank two glasses of beer.

DIAGNOSIS OF RDMEDY.—Usually when the symptoms return in a modified form a different remedy is indicated, the new symptoms being especially characteristic. In the present case, however, no remedy suited so well as *kali c.* and as the relapse seemed to be not so much a new phase of the disease as a checking of the action of the remedy by the beer (one of the worst things a rheumatic patient can take), I repeated the dose of *kali c.* 3 C. M.

Sept. 13th. Only a little of the tingling yesterday; less shooting; some dull pain still, it did not wake him at night; stomach much less tender; less irritable; less pain on deep inspiration.

Sept. 21st. No more tingling; dull pain still about heart, with occasional shooting; stomach and disposition natural; less pain on deep inspiration; at times rheumatic pain in left foot.

Sept. 28th. Took a Turkish bath on 26th, and enjoyed it; felt very well all last week; no tingling this week; no pain in legs for nearly a week.

Oct. 2d. Feels quite well. On Sept. 29th walked thirteen miles, and on 30th, twenty miles without extra fatigue. Feb. 19th, 1879. Has remained quite well in spite of the severe winter. All the time he was under my care he was compelled to continue his work, out-door collection for a public company.

COMMENTS: (1). After the first dose, the pain at the heart, which used to come on at night between 3 and 4 A.M., reached its climax between 7 and 8 P.M. and was less severe; this *postpone-*

*ment, combined with an amelioration, of a paroxysm* is a frequent sign of improvement.

(2). As the heart symptoms improved, the old trouble in foot returned; the metastasis of a symptom to a less important part or organ and the bringing back of old symptoms (with the amel. of the more recent), is also a sign of improvement.

(3). The most recent symptoms disappeared first, then the older ones, just as Hahnemann teaches; if they disappear in any other order, the cure will prove temporary only.

(4). The extended duration of action of *kali carb.* in a high potency is shown in this case; the first dose acted *curatively* from July 22d, to Sept. 10th; even apparently producing new symptoms, toward the end of that time.

Some cases require a frequent repetition of the dose and at short intervals; others need only the one single dose; this depends, in some degree, upon the homœopathicity of the remedy and also upon the freedom from external disturbing circumstances. In any case this rule is plain—*never repeat the dose as long as the patient shows a decided and progressive improvement;* even when the improvement in chronic cases appears to cease, it is better practice to wait awhile before changing remedy or repeating the dose, as periodical exacerbations of symptoms occur almost invariably during the process of a cure.

